# Characteristics, efficacy, and prognosis analysis of newly diagnosed marginal zone lymphoma

**DOI:** 10.3389/fimmu.2024.1466859

**Published:** 2024-09-23

**Authors:** Haotian Wang, Ying Zhang, Zhaoxia Li, Ou Bai

**Affiliations:** Department of Hematology, The First Hospital of Jilin University, ChangChun, Jilin, China

**Keywords:** lymphoma, marginal zone lymphoma, rituximab, obinutuzumab, survival analysis

## Abstract

**Objective:**

To retrospectively analyze the characteristics of newly diagnosed marginal zone lymphoma (MZL) patients, evaluate the efficacy of different treatment regimens, and explore prognostic factors in the era of immunotherapy.

**Methods:**

We reviewed the clinical data of newly diagnosed MZL patients treated at the Department of Hematology, The First Hospital of Jilin University, from October 2013 to October 2023. Survival differences between groups were analyzed using the log-rank test, and prognostic factors were identified.

**Results:**

A total of 265 newly diagnosed MZL patients were included, with a median age of 59 years (range 22-90). The most common pathological type was mucosa-associated lymphoid tissue (MALT) lymphoma, accounting for 66.0% of cases. Among the 147 MZL patients included in the efficacy analysis, the median follow-up was 43.4 months. Both the median progression-free survival (PFS) and overall survival (OS) were not reached. The 5-year PFS and OS rates were 76.0% and 86.6%, respectively. Patients who achieved complete response (CR) after induction therapy had significantly better PFS (*P*=0.0045), OS (*P*<0.001), and time to next treatment (TTNT) (*P*=0.0045) compared to those who did not achieve CR. A subgroup analysis was conducted on 51 MZL patients with high tumor burden who received ≥4 cycles of treatment. It was found that the CR rate (CRR) in patients receiving obinutuzumab (G) ± chemotherapy was significantly higher than in those receiving rituximab (R) ± chemotherapy (93.8% *vs*. 48.6%, *P*=0.002). Multivariate analysis revealed that disease progression or death within 24 months of initial treatment (POD24) was an independent risk factor affecting OS (*P*<0.001). Patients who experienced POD24 had a median survival of only 19.7 months, with a 3-year OS rate of just 37.6%, whereas those without POD24 had a 3-year OS rate of 97.3%.

**Conclusion:**

MZL is predominantly seen in middle-aged and elderly patients and is a specific indolent B-cell lymphoma, with MALT lymphoma being the most common subtype. Achieving CR after induction therapy significantly prolongs survival in MZL patients. Compared to R ± chemotherapy, G ± chemotherapy achieves a higher CRR in high tumor burden MZL patients. In the era of immunotherapy, POD24 is an independent prognostic factor for MZL.

## 1 Introduction

Marginal zone lymphoma (MZL) is a group of B-cell lymphomas originating from the marginal zone of lymphoid follicles, accounting for 5-15% of all non-Hodgkin lymphomas (NHL) (1, 2). MZL is considered an indolent NHL (iNHL). The World Health Organization (WHO) classifies MZL into three subtypes: extranodal MZL of mucosa-associated lymphoid tissue (MALT), splenic MZL (SMZL), and nodal MZL (NMZL) (3). In the United States, MALT lymphoma is the most common subtype, comprising 61% of all MZL cases, followed by NMZL (30%) and SMZL (9%) (4). The median survival for MZL exceeds 10 years, with varied prognoses across different pathological subtypes. MALT lymphoma has the best prognosis, with a 5-year relative survival rate of 93.8%, compared to 85.3% for SMZL and 82.8% for NMZL (4). The treatment strategies for MZL are tailored to the individual patient and include watchful waiting, anti-infective therapy, surgical resection, radiotherapy, chemotherapy, and immunochemotherapy (5, 6). Currently, there is no international consensus on the preferred first-line treatment for MZL. However, the use of anti-CD20 monoclonal antibody rituximab (R) ± chemotherapy has become widely accepted in clinical practice. Common regimens include R combined with chlorambucil, R-CHOP (rituximab, cyclophosphamide, doxorubicin, vincristine, prednisone), R-CVP (rituximab, cyclophosphamide, vincristine, prednisone), and BR (rituximab, bendamustine) (6–11). These R ± chemotherapy regimens achieve an overall response rate (ORR) of 81%, with 4-year PFS and OS rates of 64.1% and 78.1%, respectively. Nevertheless, some patients exhibit resistance, either not responding to R treatment or experiencing rapid disease progression post-treatment (12, 13). Encouragingly, the advent of obinutuzumab (GA101; G) offers new possibilities for improving MZL treatment outcomes. G is a next-generation, humanized, glycoengineered type II anti-CD20 monoclonal antibody, characterized by enhanced antibody stability, superior antibody-dependent cellular cytotoxicity (ADCC), direct cell death induction, and faster target binding kinetics (14, 15). G has demonstrated efficacy in various B-cell NHL types, such as follicular lymphoma (FL) and chronic lymphocytic leukemia/small lymphocytic lymphoma (CLL/SLL) (16, 17). Notably, the GALLIUM study, which included 1202 patients, aimed to compare the efficacy of G-chemotherapy versus R-chemotherapy as first-line treatment for FL (16). With a median follow-up of 41 months, the G-chemotherapy group showed a 46.0% reduction in the risk of disease progression or death within 24 months of initial treatment (POD24) compared to the R-chemotherapy group (18, 19). Consequently, in June 2021, the National Medical Products Administration (NMPA) of China approved G for adult FL patients. Given the similarities between MZL and FL, both being highly heterogeneous and currently incurable iNHLs, G, with its remarkable mechanism of action and pharmacological profile, has the potential to become a novel therapeutic option for MZL patients.

To explore real-world efficacy, this study aims to retrospectively analyze 265 newly diagnosed MZL cases treated at the Department of Hematology, The First Hospital of Jilin University, from October 2013 to October 2023. The study will summarize the characteristics of MZL, evaluate the effectiveness of different treatment regimens, and investigate prognostic factors in the era of immunotherapy.

## 2 Materials and methods

### 2.1 Study subjects

This study included newly diagnosed MZL patients treated at the Department of Hematology, The First Hospital of Jilin University, from October 2013 to October 2023. The diagnostic criteria were based on the 2016 WHO classification of lymphoid neoplasms (20). Inclusion Criteria: Patients of any gender, aged 18 years or older. Pathologically confirmed diagnosis of MZL. No prior treatment history. Exclusion Criteria: Patients with no clear indication for treatment. Patients who received only anti-infective therapy, surgery, or radiotherapy. Patients who received only chemotherapy. Patients who underwent fewer than two treatment cycles. Patients with a history of malignancies. Patients lost to follow-up. This study was conducted in accordance with the guidelines of the Declaration of Helsinki and was approved by the Ethics Committee of The First Hospital of Jilin University.

### 2.2 Study data

#### 2.2.1 Relevant data

The study collected various types of data, including: General Information: Name, gender, age, etc. Clinical Data: B symptoms, ECOG performance status, marginal zone lymphoma international prognostic index (MZL-IPI), etc. Pathological Results: Pathological subtype, immunohistochemistry, etc. Laboratory Tests: Complete blood count, blood biochemistry, lactate dehydrogenase (LDH), β2-microglobulin (β2-MG), bone marrow biopsy, etc. Imaging Studies: CT, PET-CT, etc. Treatment Regimens: Rituximab (R) ± chemotherapy, obinutuzumab (G) ± chemotherapy. Treatment Efficacy: Complete response (CR), partial response (PR), overall response rate (ORR), progression-free survival (PFS), overall survival (OS), time to next treatment (TTNT) etc.

#### 2.2.2 Disease-related concepts

##### 2.2.2.1 Treatment indications for MZL patients (referencing FL)

Availability of suitable clinical trials. Presence of any discomfort affecting normal work and life. End-organ function impairment. Lymphoma-induced cytopenias due to bone marrow involvement. Bulky disease (referencing GELF criteria). Persistent or rapidly progressing disease.

##### 2.2.2.2 GELF high tumor burden criteria

Involvement of ≥3 lymph node regions with diameters ≥3 cm. Any lymph node or extranodal tumor mass with a diameter ≥7 cm. B symptoms. Splenomegaly. Presence of pleural effusion or ascites. White blood cell count <1.0×10^9/L or platelet count <100×10^9/L. Malignant cell count >5.0×10^9/L.

##### 2.2.2.3 MZL-IPI

LDH, hemoglobin levels, platelet count, absolute lymphocyte count, MZL subtype.

### 2.3 Efficacy evaluation

Efficacy was assessed using the revised Lugano classification criteria from the 2014 Lugano Conference, categorizing responses into CR, PR, stable disease (SD), progressive disease (PD), and ORR (21).

### 2.4 Statistical methods

This study utilized SPSS Statistics 27.0 and R version 4.4.1 for data analysis. Continuous variables following a normal distribution were described using mean ± standard deviation and compared between groups using independent sample t-tests. Categorical variables were described using frequencies and percentages, and differences between groups were assessed using the chi-square test or Fisher’s exact test. Survival curves were plotted using GraphPad Prism 10, and differences in survival between groups were analyzed using the log-rank test. Univariate and multivariate prognostic analyses were performed using the Cox proportional hazards model. A *P* < 0.05 was considered statistically significant. Factors with a *P* < 0.05 were used to construct a nomogram using R.

## 3 Results

### 3.1 Characteristic analysis

**Figure 1:**
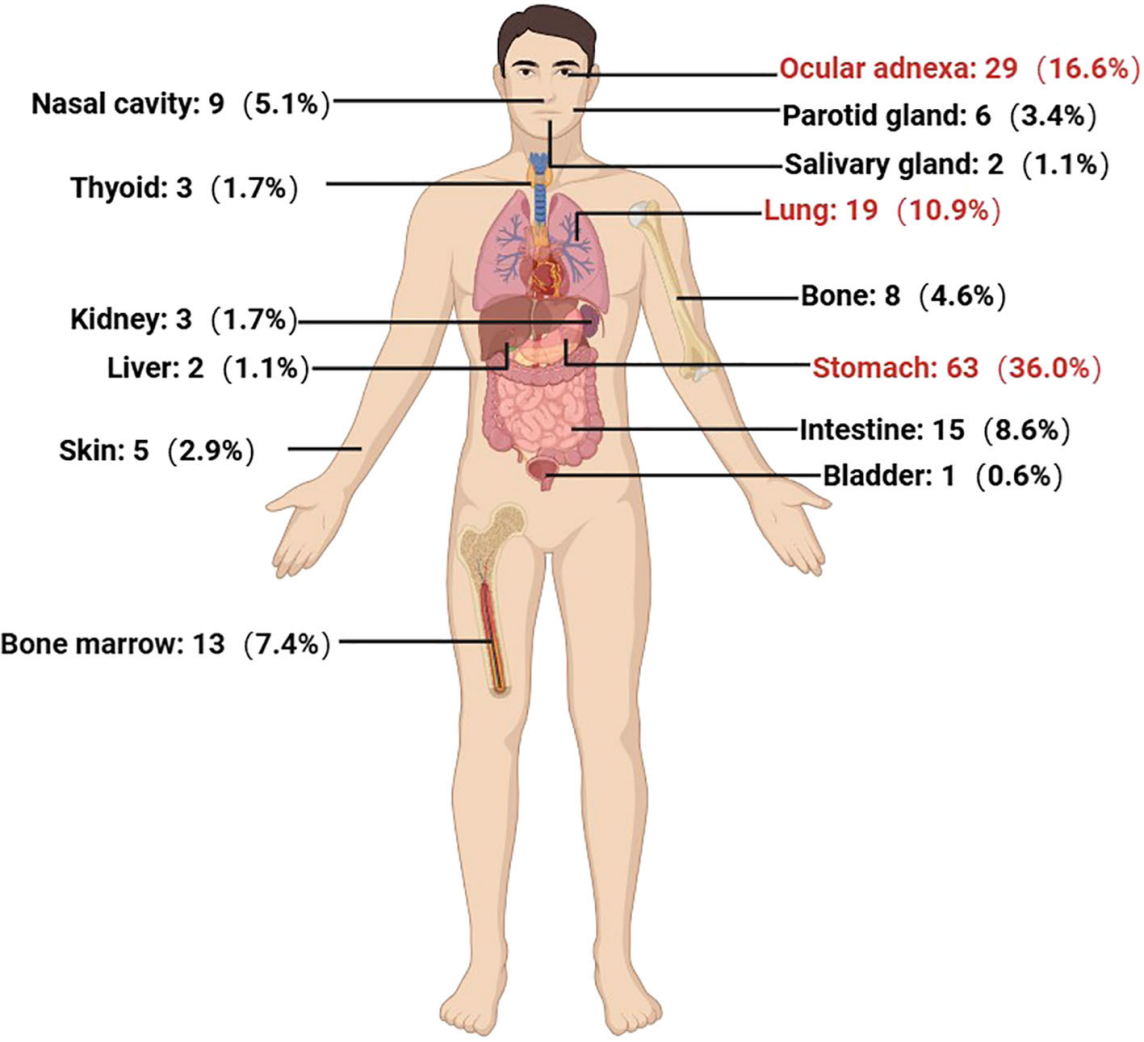
Distribution of extranodal involvement in 175 cases of MALT lymphoma.

From October 2013 to October 2023, a total of 265 newly diagnosed MZL patients were treated at our center. The median age at diagnosis was 59 years (range: 22-90 years), with a male-to-female ratio of 1.10:1. The most common pathological subtype was MALT lymphoma, accounting for 66.0% (n=175) of cases, followed by NMZL (n=42, 15.8%) and SMZL (n=40, 15.1%). Among the 191 MALT lymphoma cases, the most frequently involved extranodal site was the stomach (n=63, 36.0%), followed by ocular adnexa (n=29, 16.6%), lungs (n=19, 10.9%), intestines (n=15, 8.6%), and bone marrow (13, 7.4%) (**Figure 1**).

At initial diagnosis, nearly half of the patients (44.9%) were in the early stages (Stage I-II). The majority of patients did not present with B symptoms (n=208, 79.1%), and most were classified into the low-to-intermediate risk group based on the MZL-IPI score (n=234, 88.3%). Laboratory findings indicated that 216 patients (81.5%) had normal LDH levels, and 189 patients (71.3%) had no bone marrow involvement. Among the 40 SMZL patients, 39 underwent bone marrow biopsy, with 92.3% (n=36) showing bone marrow involvement, which was significantly higher than the rates in MALT lymphoma and NMZL (7.4% and 43.9%, respectively) (*P* < 0.001). Helicobacter pylori infection was detected in 29 patients (10.9%), with 96.6% (28/29) of these cases being MALT lymphoma, and 60.7% (17/28) involving the gastrointestinal tract. Additionally, 29 patients (10.9%) had hepatitis B virus infection/carrier status, and 16 patients (6.0%) had Epstein-Barr virus infection (**Table 1**).

**Table 1:** Baseline characteristics of 265 MZL patients.

Characteristics		n (%)
Age	<60 years	134 (50.6)
	≥60 years	131 (49.4)
Sex	Male	139 (52.5)
	Female	126 (47.5)
Pathological subtype	MALT	175(66.0)
	SMZL	40 (15.1)
	NMZL	42 (15.8)
	MZL (unclassified)	8 (3.0)
Ann Arbor stage	I-II	119 (44.9)
	III-IV	135 (50.9)
	Missing	11 (4.2)
B symptoms	No	208 (79.1)
	Yes	55 (20.9)
MZL-IPI	0	106 (40.0)
	1-2	128 (48.3)
	3-5	25 (9.4)
	Missing	6 (2.3)
ECOG	<2	224 (84.5)
	≥2	41 (15.5)
LDH	Normal	216 (81.5)
	Raise	43 (16.2)
	Missing	6 (2.3)
β2-MG	Normal	48 (18.1)
	Raise	255 (78.1)
	Missing	10 (3.8)
Bone marrow involvement	No	189 (71.3)
	Yes	71 (26.8)
	Missing	5 (1.9)
Infection	Helicobacter pylori	29 (10.9)
	Hepatitis B virus	29 (10.9)
	Epstein-Barr virus	16 (6.0)

MALT, mucosa-associated lymphoma tissue; SMZL, splenic marginal zone lymphoma; NMZL, nodal marginal zone lymphoma; MZL, marginal zone lymphoma; IPI, international prognostic index; ECOG, Eastern Cooperative Oncology Group; LDH, lactate dehydrogenase; β2-MG, β2-microglobulin.

### 3.2 Efficacy analysis

**Figure 2:**
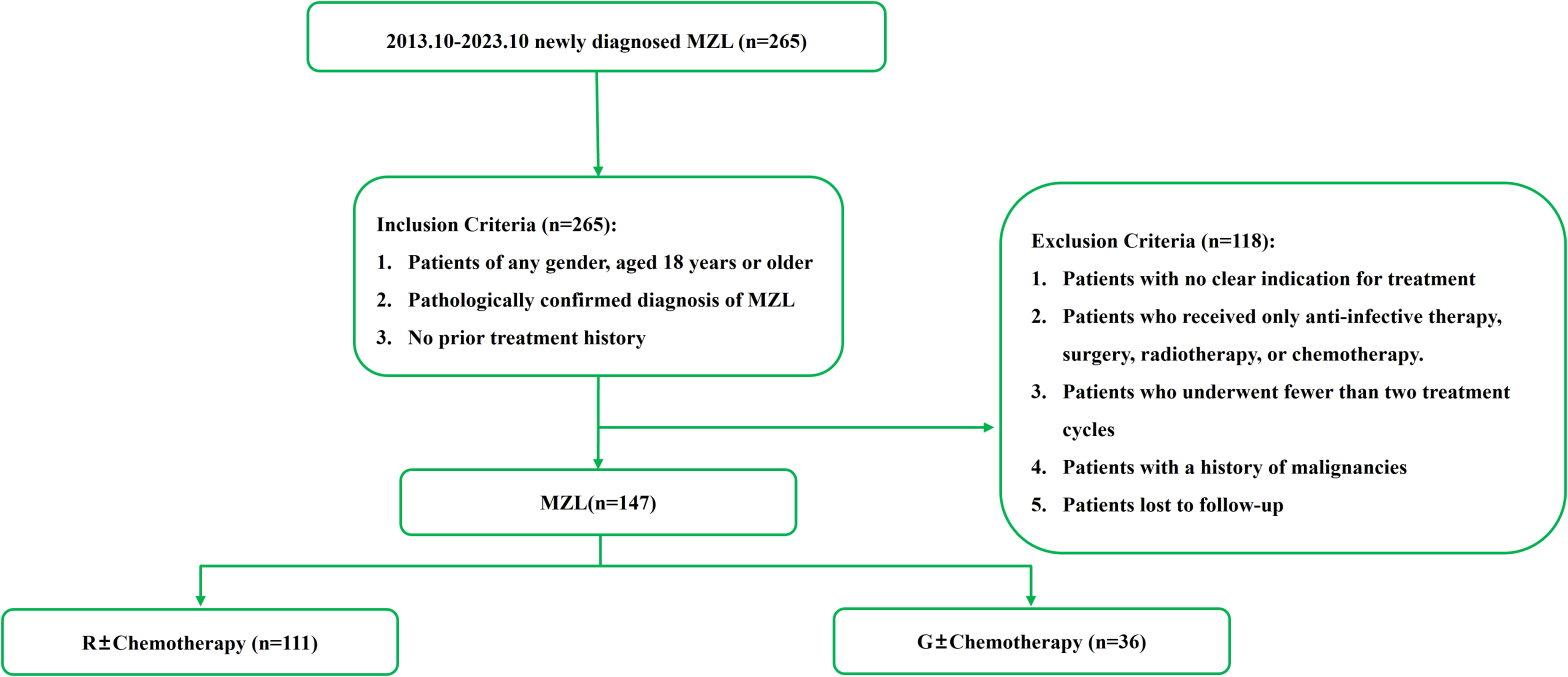
Inclusion of cases for efficacy and prognosis analysis in this study.

**Figure 3:**
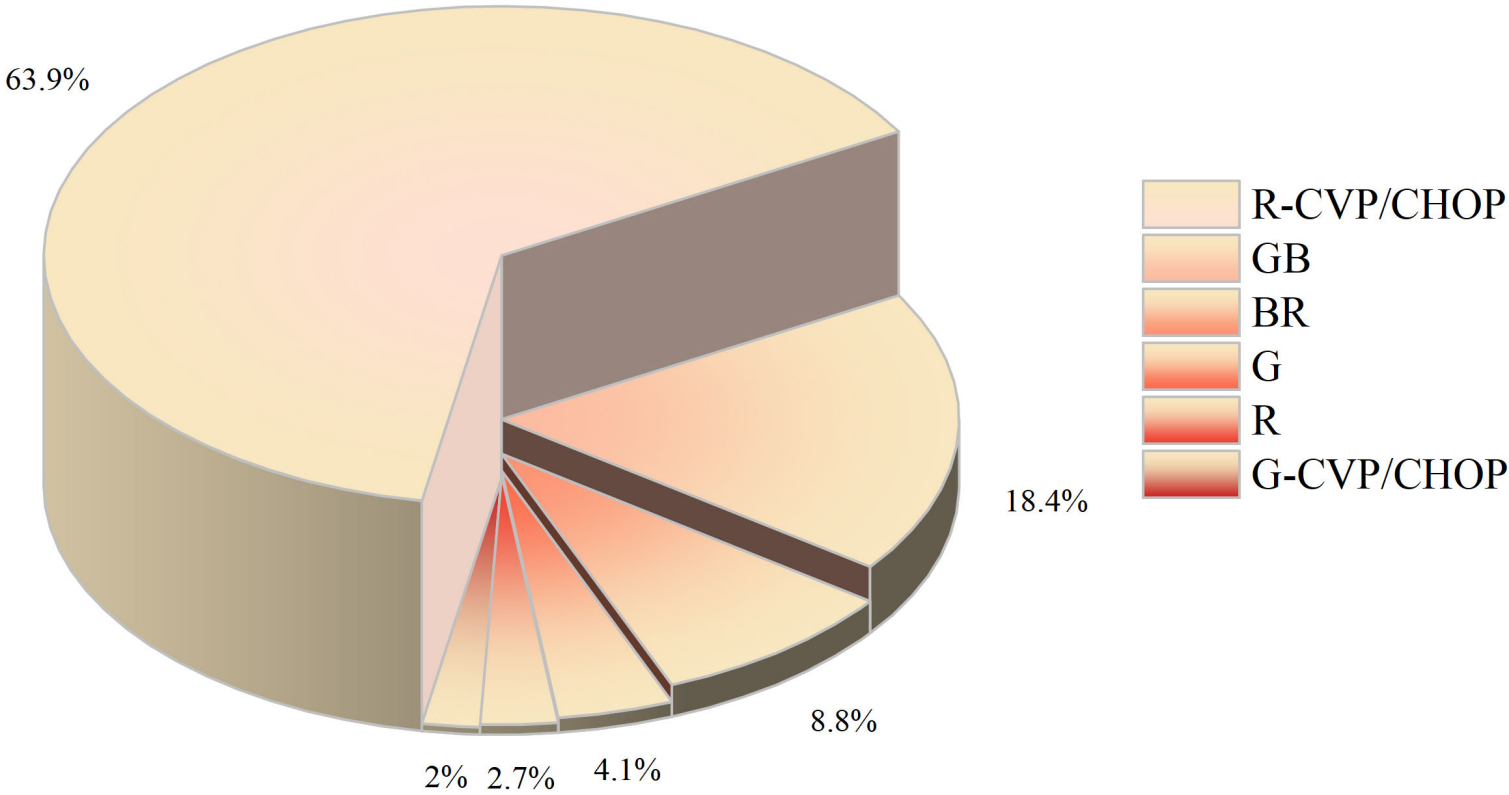
Different induction therapy regimens for first-line treatment of MZL.

**Figure 4:**
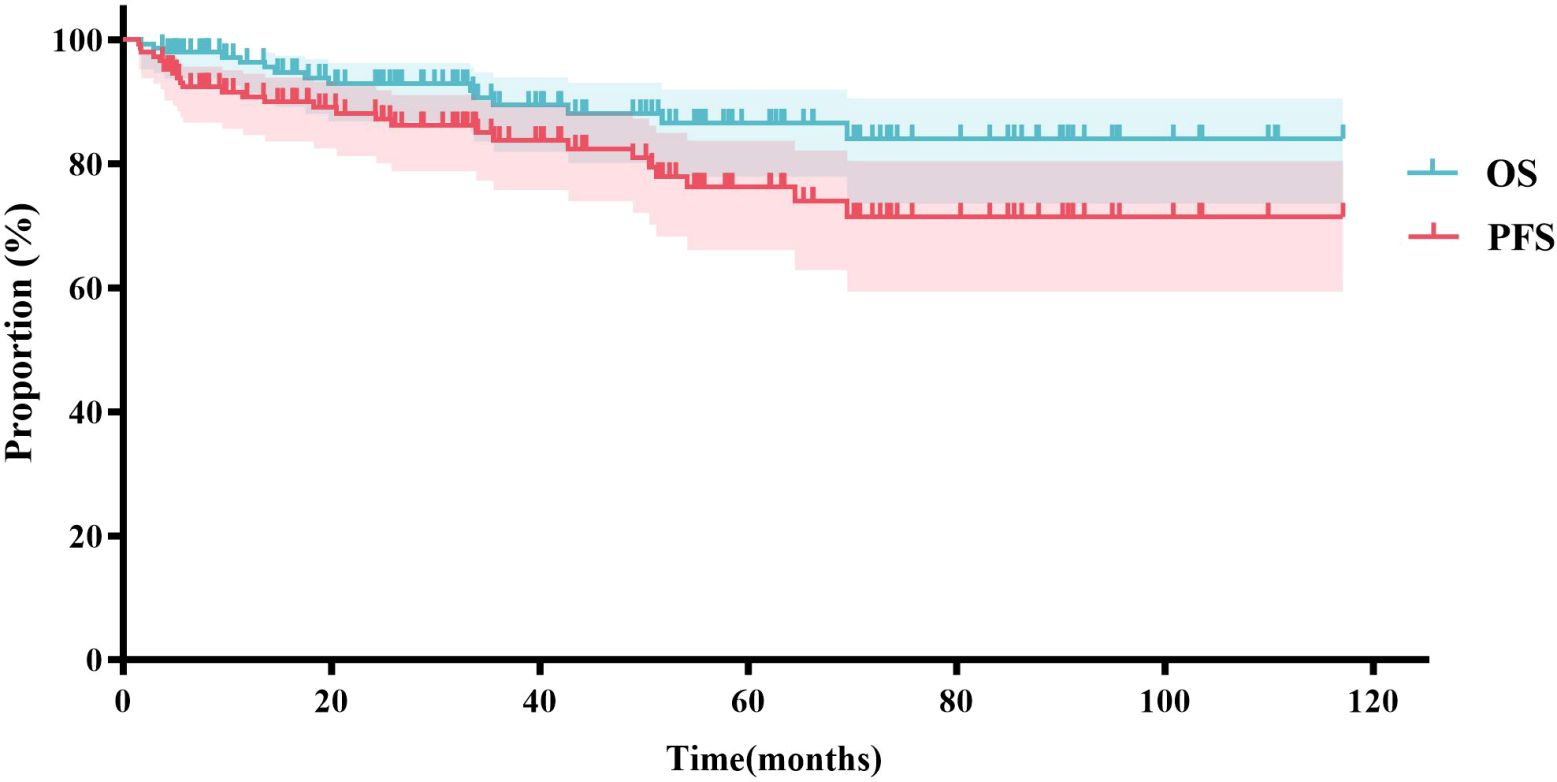
Efficacy analysis of 147 MZL cases.

Based on the inclusion and exclusion criteria (**Figure 2**), a total of 147 MZL patients were included in the final efficacy analysis. Following induction therapy, 83 patients (56.5%) achieved CR, with an ORR of 92.5%. No significant difference in CR rates (CRR) was observed between patients receiving R or G induction therapy (55.8% *vs.* 58.3%, *P*=0.8483). Additionally, there was no significant difference in CRR between the same chemotherapy regimens combined with R or G (R-CVP/CHOP *vs*. G-CVP/CHOP: 56.4% *vs.* 66.7%, *P*>0.999; BR *vs.* GB: 61.5% *vs.* 66.7%, *P*>0.999) (**Figure 3**, **Table 2**). With a median follow-up of 43.4 months, neither the median PFS nor the OS was reached. In the overall analysis, the 3-year and 5-year PFS rates were 83.8% and 76.3%, respectively, while the 3-year and 5-year OS rates were 89.5% and 86.6%, respectively (**Figure 4**).

**Table 2:** Short-term efficacy of different induction therapy regimens.

n (%)	G-CVP/CHOP (n=3)	GB (n=27)	G (n=6)	G±chemo (n=36)	R-CVP/CHOP (n=94)	BR (n=13)	R (n=4)	R±chemo (n=111)
CR	2 (66.7)	18 (66.7)	1 (16.7)	21 (58.3)	53 (56.4)	8 (61.5)	1 (25.0)	62 (55.8)
PR	1 (33.3)	9 (33.3)	5 (83.3)	15 (41.7)	31 (33.0)	5 (38.5)	2 (50.0)	38 (34.2)
SD/PD	0 (0)	0 (0)	0 (0)	0 (0)	10 (10.6)	0 (0)	1 (25.0)	11 (9.9)

G, obinutuzumab; R, rituximab; CVP, cyclophosphamide, vincristine, prednisone; CHOP, cyclophosphamide, doxorubicin, vincristine, prednisone; B, bendamustine; chemo, chemotherapy; CR, complete response; PR, partial response; SD, stable disease; PD, progressive disease.

**Figure 5:**
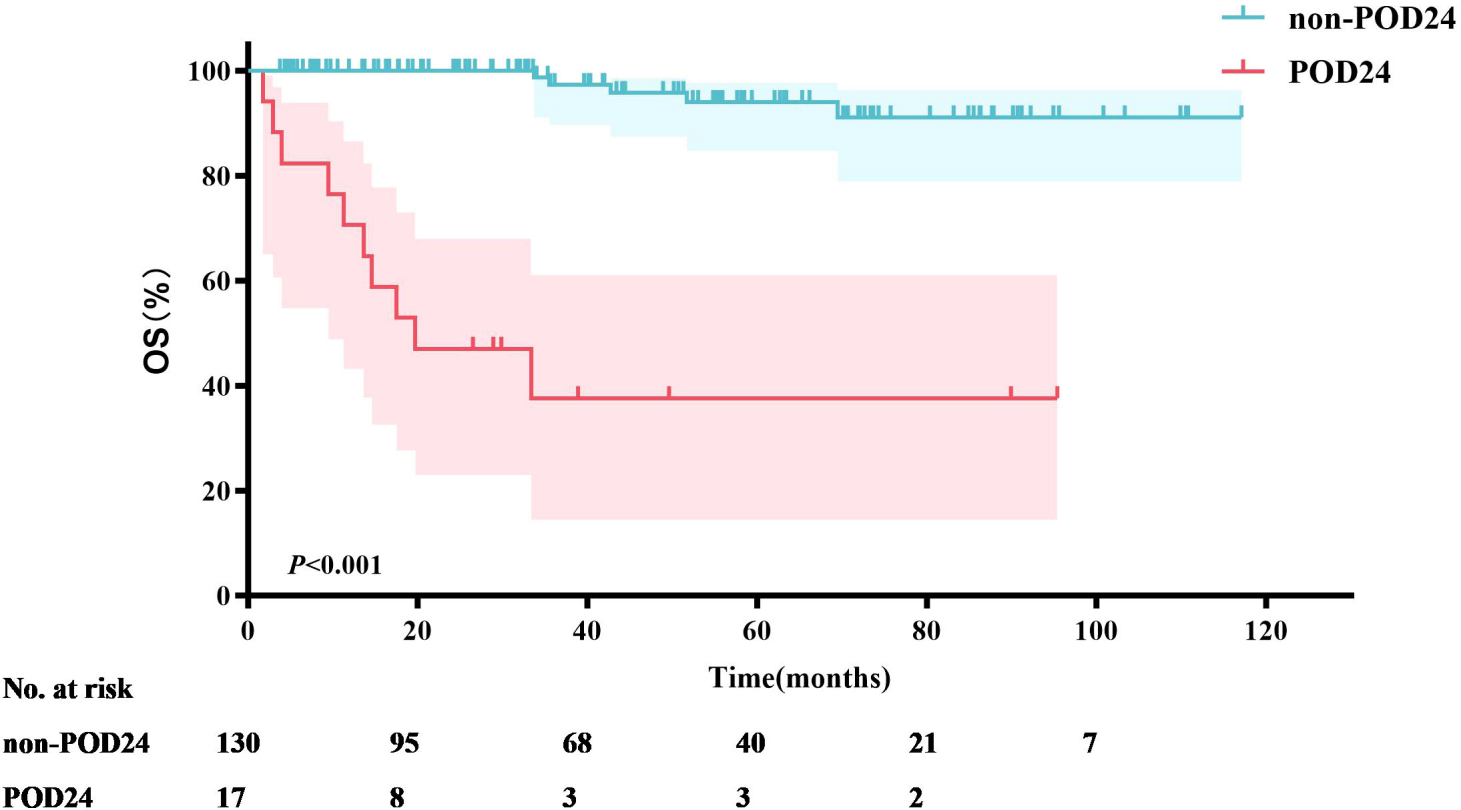
Survival analysis of POD24 *vs*. non-POD24 (OS).

Seventeen MZL patients (11.6%) experienced POD24. Survival analysis was performed based on whether patients experienced POD24. The results showed that the median OS for patients with POD24 was only 19.7 months. In contrast, the median OS for patients without POD24 was not reached (*P*<0.001) (**Figure 5**), with a 5-year OS rate of 94.0%.

**Figure 6:**
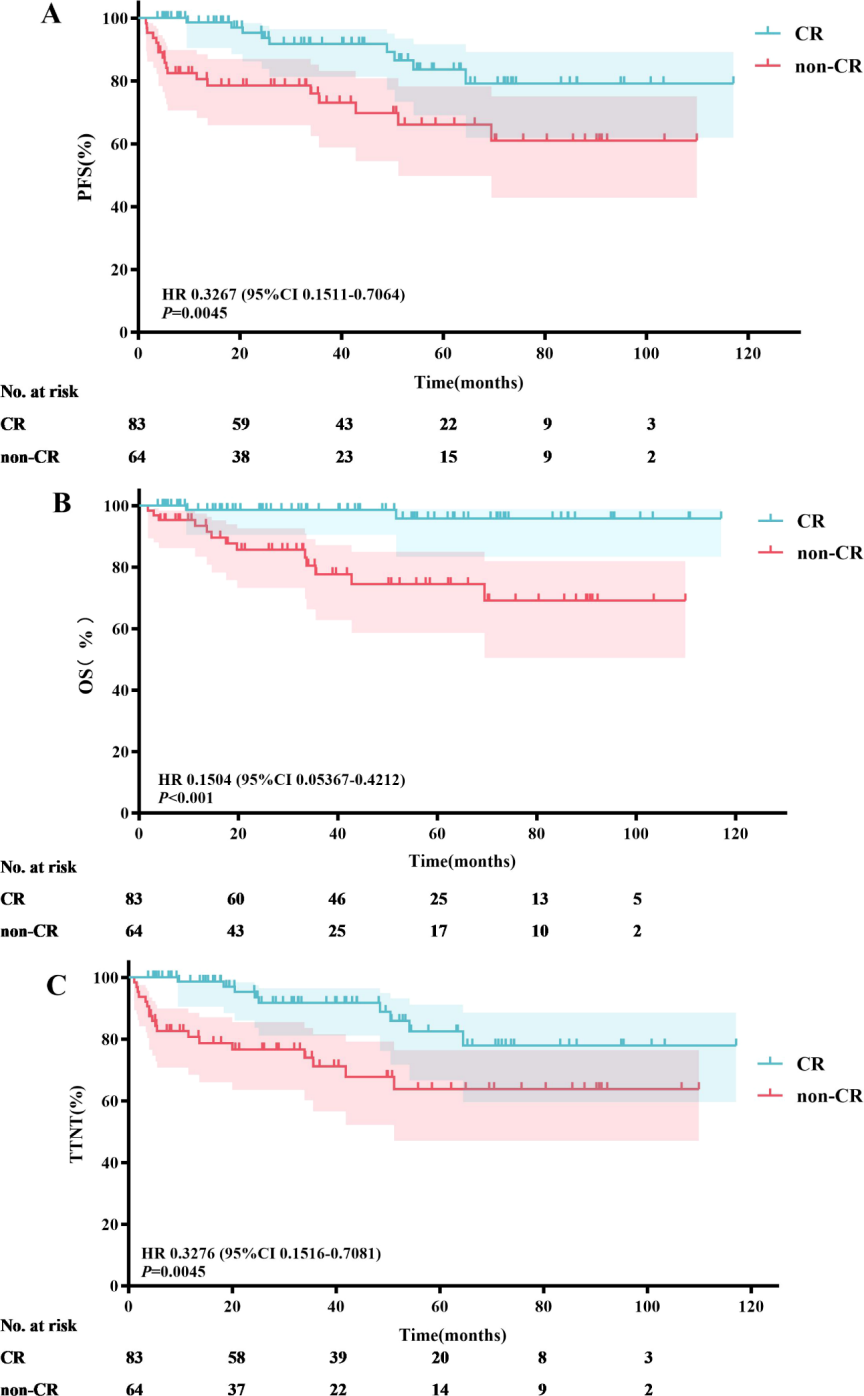
Efficacy analysis of patients achieving CR *vs*. not achieving CR after induction therapy: PFS **(A)**, OS **(B)** and TTNT **(C)**.

A total of 83 patients (56.7%) achieved CR following induction therapy. A subgroup analysis was conducted based on whether patients achieved CR after induction therapy. The results indicated that the median PFS, OS, and TTNT were not reached in either group. In the CR group, the 3-year and 5-year PFS rates were 91.8% and 83.6%, respectively; the 3-year and 5-year OS rates were 98.6% and 95.9%, respectively; and the 3-year and 5-year TTNT rates were 91.7% and 82.5%, respectively. In contrast, the non-CR group had 3-year and 5-year PFS rates of 73.2% and 66.2%, respectively; 3-year and 5-year OS rates of 77.7% and 74.5%, respectively; and 3-year and 5-year TTNT rates of 71.2% and 63.8%, respectively. Patients who achieved CR after induction therapy had significantly longer PFS (*P*=0.0045), OS (*P*<0.001), and TTNT (*P*=0.0045) compared to those who did not achieve CR (**Figure 6**).

A subgroup analysis was conducted comparing G ± chemotherapy and R ± chemotherapy. There was a significant difference in baseline characteristics between the two groups, particularly in the presence of high tumor burden (*P*=0.003) (**Table 3**). Among the 63 MZL patients with high tumor burden, 51 patients (35 in the R ± chemotherapy group and 16 in the G ± chemotherapy group) received four or more treatment cycles. A short-term efficacy analysis was performed based on the different treatment regimens for these patients. The results showed that after four treatment cycles, 15 patients (93.8%) in the G ± chemotherapy group achieved CR, compared to 17 patients (48.6%) in the R ± chemotherapy group, with a significant difference between the two groups (*P*=0.002). However, among MZL patients without high tumor burden, there was no significant difference in the CRR between the G ± chemotherapy and R ± chemotherapy groups (54.5% *vs.* 63.4%, *P*=0.740).

**Table 3:** Baseline characteristics of MZL patients treated with different anti-CD20 monoclonal antibodies.

Characteristics			R±chemotherapy (n=111)	G±chemotherapy (n=36)	*χ^2^*	*P*
Sex					0.055	0.814
	Male		58 (52.3%)	18 (50.0%)		
	Female		53 (47.7%)	18 (50.0%)		
Age					0.055	0.814
	<60 years		58 (52.3%)	18 (50.0%)		
	≥60 years		53 (47.7%)	18 (50.0%)		
Ann Arbor stage					0.625	0.429
	I-II		42 (37.8%)	11 (30.6%)		
	III-IV		69 (62.2%)	25 (69.4%)		
MZL-IPI					0.634	0.811
	0		41 (36.9%)	11 (30.6%)		
	1-2		60 (54.1%)	21 (58.3%)		
	3-5		10 (9.0%)	4 (11.1%)		
B symptoms					3.244	0.072
	no		90 (81.1%)	24 (66.7%)		
	yes		21 (18.9%)	12 (33.3%)		
ECOG					0.006	0.940
	0-1		87 (78.4%)	28 (77.8%)		
	≥2		24 (21.6%)	8 (22.2%)		
High tumor burden					8.611	0.003
	no		71 (64.0%)	13 (36.1%)		
	yes		40 (36.0%)	23 (63.9%)		
INV-assessed response					4.195	0.128
	CR		62 (55.9%)	21 (58.3%)		
	PR		38 (34.2%)	15 (41.7%)		
	SD/PD		11 (9.9%)	0 (0)		

MZL, marginal zone lymphoma; MZL-IPI, MZL-international prognostic index; ECOG, Eastern Cooperative Oncology Group; G, obinutuzumab; R, rituximab; INV, investigator; CR, complete response; PR, partial response; SD, stable disease; PD, progressive disease.

### 2.3 Prognostic analysis

**Figure 7:**
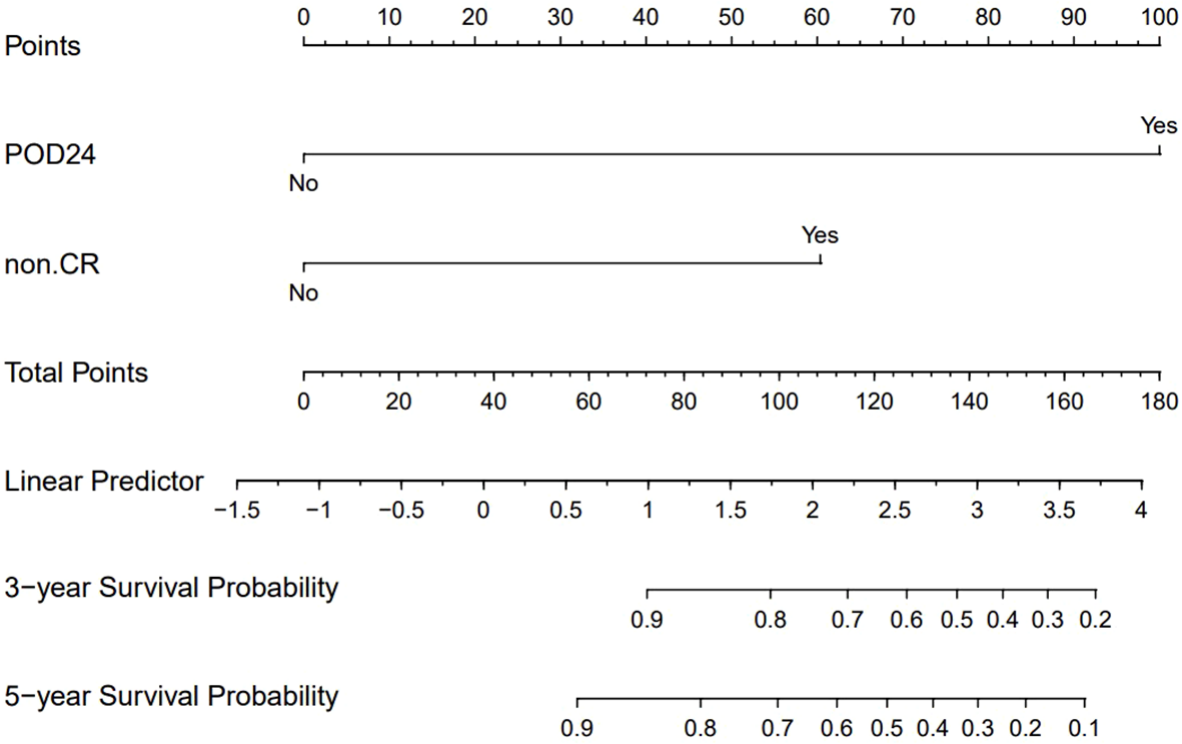
OS nomogram prognostic model.

After conducting univariate and multivariate analyses on 147 MZL patients, the results indicated that failure to achieve CR following induction therapy (HR: 3.250, 95% CI=1.409-7.500, *P*=0.006) was an independent factor affecting PFS. Additionally, failure to achieve CR after induction therapy (HR: 5.1766, 95% CI=1.075-24.934, *P*=0.040) and the occurrence of POD24 (HR: 22.544, 95% CI=6.390-79.541, *P*<0.001) were independent factors influencing OS (**Supplementary Data**, **Supplementary Tables S1**, **S2**). Based on these results, we assigned values to factors with *P*<0.05 and summed the scores of each parameter to obtain a total score. This total score was then converted to OS using a conversion relationship, leading to the construction of a nomogram related to OS. The results demonstrated that the occurrence of POD24 had the greatest impact on prognosis (**Figure 7**). Furthermore, the effectiveness of the model was evaluated using ROC curve analysis. The results showed that the AUC for the OS prediction model was 0.938 at 3 years and 0.843 at 5 years, indicating that the nomogram had good discriminative ability (**Supplementary Data**, **Supplementary Figure S1**). The calibration curve showed slight bias (**Supplementary Data**, **Supplementary Figures S2**, **S3**).

**Figure 8:**
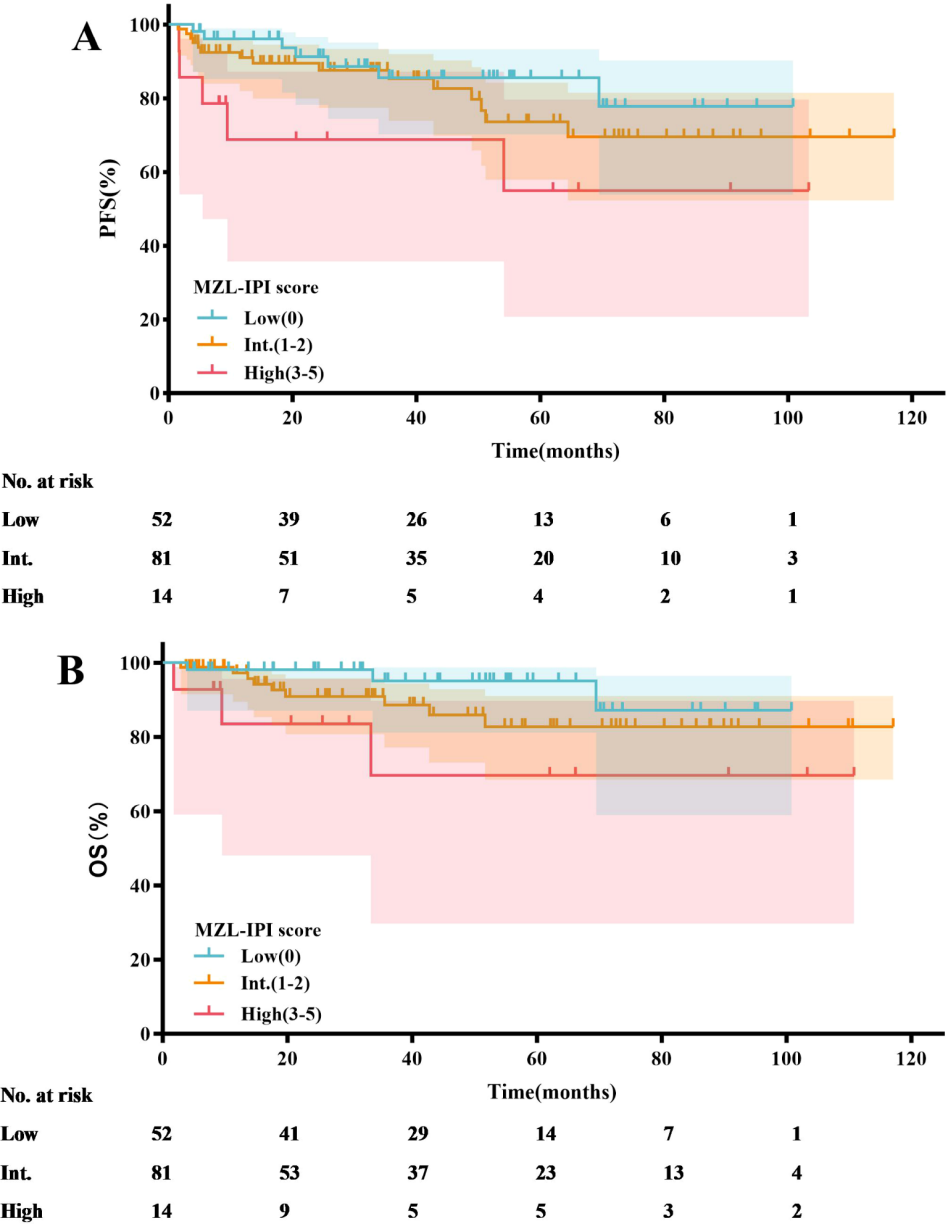
MZL-IPI risk stratification analysis: PFS **(A)**, OS **(B)**.

Based on the MZL-IPI score, patients were classified into low-risk, intermediate-risk, and high-risk groups. The low-risk group included 52 patients (35.4%), the intermediate-risk group included 81 patients (55.1%), and the high-risk group included 14 patients (9.5%). The 5-year PFS rates for the low-risk, intermediate-risk, and high-risk groups were 85.5%, 73.6%, and 55.0%, respectively. Similarly, the 5-year OS rates were 95.1%, 82.8%, and 69.6%, respectively. Statistical analysis revealed that, compared to the low-risk group, the high-risk group had significantly shorter PFS (*P*=0.025) and OS (*P*=0.040) (**Figure 8**, **Table 4**).

**Table 4:** MZL-IPI risk stratification analysis.

(A) PFS				
Group	n (%)	5-yr PFS%	HR (95%CI)	*P*
Low (0)	52 (35.4)	85.5		
Int. (1-2)	81 (55.1)	73.6	1.520 (0.6511-3.546)	0.333
High (3-5)	14 (9.5)	55	5.529 (1.234-24.76)	0.025
High *vs.* Int.			2.610 (0.728-9.358)	0.141
(B) OS				
Group	n (%)	5-yr OS%	HR (95%CI)	*P*
Low (0)	52 (35.4)	95.1		
Int. (1-2)	81 (55.1)	82.8	2.031 (0.6451-6.396)	0.226
High (3-5)	14 (9.5)	69.6	8.940 (1.104-72.39)	0.04
High *vs.* Int.			2.835 (0.5304-15.16)	0.223

MZL-IPI, marginal zone lymphoma-international prognostic index; PFS, progression-free survival; OS, overall survival; Int., Intermediate.

### 3.4 Safety analysis

In this retrospective study, we observed a relatively low number of adverse events (AEs) across the treatment groups. Due to the limited sample size and the infrequency of AEs, the study did not achieve sufficient statistical power to conduct a comprehensive analysis of safety differences. Therefore, we did not perform a significance comparison of AE incidence rates between the different treatment groups.

Despite a higher proportion of infusion reactions in the G± chemotherapy group (41.7%) compared to the R± chemotherapy group (16.2%), no patients discontinued treatment as a result (**Table 5**). In the R± chemotherapy group, 31.5% of patients experienced grade 3-4 adverse events, with the most common hematologic AEs being leukopenia and neutropenia (both occurring in 17 patients, 15.3%). The most common non-hematologic AE was pneumonia (9 patients, 8.1%). In contrast, 52.8% of patients in the G± chemotherapy group experienced grade 3-4 AEs, with leukopenia being the most common hematologic AE (16 patients, 44.4%) and pneumonia being the most common non-hematologic AE (8 patients, 22.2%). Additionally, two patients in the G± chemotherapy group died from COVID-19 infections within 1-6 months after completing treatment.

**Table 5:** AEs by chemotherapy arm.

n (%)		G-CVP/CHOP (n=3)	GB (n=27)	G (n=6)	G±chemo (n=36)	R-CVP/CHOP (n=94)	BR (n=13)	R (n=4)	R±chemo (n=111)
Infusion reactions		2 (66.7)	11 (40.7)	2 (33.3)	15 (41.7)	15 (16.0)	2 (15.4)	1 (25.0)	18 (16.2)
Grade 3-4AEs		3 (100)	14 (51.9)	2 (33.3)	19 (52.8)	29 (30.9)	6 (46.2)	0 (0)	35 (31.5)
	Hematological	3 (100)	12 (44.4)	1 (16.7)	13 (36.1)	20 (21.3)	6 (46.2)	0 (0)	26 (23.4)
	Non-hematological	0 (0)	6 (22.2)	2 (33.3)	8 (22.2)	11 (11.7)	3 (23.1)	0 (0)	14 (12.6)

AEs, adverse events; G, obinutuzumab; R, rituximab; CVP, cyclophosphamide, vincristine, prednisone; CHOP, cyclophosphamide, doxorubicin, vincristine, prednisone; B, bendamustine; chemo, chemotherapy.

## 4 Discussion

### 4.1 Characteristic analysis

MZL is the second most common iHNL after FL, accounting for 5-15% of all NHL cases (1). MZL originates from the marginal zone of lymphoid follicles and can occur in mucosa-associated lymphoid tissue, the spleen, and lymph nodes. MZL is highly heterogeneous, and the WHO classifies it into three subtypes: MALT lymphoma, SMZL, and NMZL (3). According to epidemiological studies in the United States, MALT lymphoma is the most common subtype of MZL, comprising 61% of cases, followed by NMZL (30%) and SMZL (9%) (4). In this retrospective study, the median age at diagnosis for 265 newly diagnosed MZL patients was 59 years (range: 22-90 years). Among these patients, 66.0% had MALT lymphoma, 15.8% had NMZL, 15.1% had SMZL, and 3.0% had unclassified MZL. Unlike previous studies, the incidence of SMZL in our study was notably higher. This discrepancy may be attributed to the following reasons: historically, the diagnosis of SMZL relied on splenic biopsy. Currently, for patients who cannot undergo splenic biopsy, diagnosis can be made based on the characteristic cytomorphology in peripheral blood or bone marrow, combined with immunophenotyping and the presence of CD20-positive sinusoidal infiltration in bone marrow pathology (22).

Most patients had low to intermediate-risk prognostic scores (88.3%), normal LDH levels (81.5%), an ECOG performance status of less than 2 (84.5%), and no B symptoms (79.1%). These factors collectively suggest that MZL is an indolent lymphoma characterized by a prolonged disease course and slow progression. In the absence of clear treatment indications, a strategy of watchful waiting is feasible (23). The extranodal involvement of MZL exhibits specificity. Current studies confirm that the most common site of extranodal involvement in MALT lymphoma is the stomach, followed by ocular adnexa and lungs, which is consistent with our findings (1, 24). In our cohort of 265 MZL patients, 71 (26.8%) had bone marrow involvement. Among these, 36 cases were SMZL, accounting for 90.0% of all SMZL cases in this study, which is significantly higher than NMZL and MALT lymphoma (43.9% and 7.4%, respectively; *P* < 0.001). This aligns with previous research (25, 26), indicating that SMZL is more prone to bone marrow involvement compared to MALT lymphoma and NMZL.

In summary, at our center, MZL predominantly affects middle-aged and elderly patients, with MALT lymphoma being the most common pathological subtype. Most patients fall into the low to intermediate-risk prognostic group, with the stomach being the most frequent site of extranodal involvement. SMZL shows a higher propensity for bone marrow involvement compared to other MZL subtypes.

### 4.2 Efficacy analysis

This study included 147 patients for efficacy analysis. After induction therapy, the ORR reached 92.5%, and the CRR exceeded 50%. Specifically, the ORR and CRR in the G± chemotherapy group were 100% and 58.3%, respectively, which are higher than the results reported in the GALLIUM study for the G-chemotherapy group (ORR of 81.8% and CRR of 17.2%) (12). These differences may be attributed to factors such as baseline patient characteristics and treatment protocols, and they may also reflect variations in the efficacy of G in different study settings. These findings highlight the potential advantages and prospects of G in the frontline treatment of MZL.

In the study by Kang et al. (8), 40 treatment-naïve MZL patients received R-CVP as first-line therapy. With a median follow-up of 38.2 months, neither median PFS nor OS was reached, with estimated 3-year PFS and OS rates of 59% and 95%, respectively. In the GALLIUM study (12), the R-chemotherapy group included 96 patients with a median follow-up of 59.3 months, reporting 4-year PFS and OS rates of 64.1% and 78.1%, respectively. The G-chemotherapy group, comprising 99 patients, demonstrated 4-year PFS and OS rates of 72.6% and 81.8%, respectively. Our study included 147 treatment-naïve MZL patients who received anti-CD20 monoclonal antibody therapy with or without chemotherapy. The median follow-up was 43.4 months, and neither median PFS nor OS was reached. The 5-year PFS and OS rates were 76.3% and 86.6%, respectively. In the R ± chemotherapy subgroup (111 patients), the 5-year PFS and OS were 76.0% and 86.9%, respectively. Compared to other studies, our R ± chemotherapy group had a larger patient cohort and a longer median follow-up than Kang et al.’s study, showing a slight advantage in PFS and OS.

It remains unclear whether the degree of disease remission influences prognosis. In this study, we performed a survival analysis on patients who achieved CR after induction therapy and found that CR significantly prolonged the survival of patients with MZL. These findings hold significant implications for clinical practice. Firstly, patients who achieved CR after induction therapy showed marked improvements in PFS, OS, and TTNT. This indicates that patients achieving CR have a clear advantage in terms of long-term survival and quality of life. However, it is important to note that while achieving CR is associated with better survival rates, this observation should not be simply interpreted as a direct causal relationship. Patients achieving CR may have inherently favorable disease biological characteristics, which contribute to their improved outcomes, rather than solely the effect of the treatment itself. Secondly, these results underscore the importance of early assessment of treatment efficacy. By adjusting treatment plans based on early assessment results, clinicians can maximize patient benefits. Early assessment not only helps identify patients who are not responding well to the current treatment regimen but also allows for the development of more individualized treatment strategies for these patients, thereby enhancing the effectiveness and safety of the treatment. Additionally, we must consider the individual differences among patients. Patients who achieve CR may possess more favorable disease characteristics, such as lower tumor burden and better biomarker status. Therefore, in clinical practice, physicians should comprehensively consider the individual characteristics and biological features of the disease in each patient to formulate the most appropriate treatment plan. In conclusion, although this study indicates that achieving CR is associated with better prognosis in MZL patients, we should interpret this result with caution and avoid simply viewing it as a causal relationship. Clinicians should strive to evaluate and optimize treatment plans throughout the treatment process to achieve the best possible long-term survival and quality of life for suitable patients. Future research should further explore the relationship between CR and prognosis and investigate additional factors influencing prognosis to guide clinical practice.

Drawing from previous research, G has shown efficacy in treating high tumor burden FL (27). In this study, we analyzed 63 patients who had a high tumor burden, of which 51 patients received at least four cycles of treatment. We compared the short-term efficacy (ORR and CRR) between patients receiving G ± chemotherapy and those receiving R ± chemotherapy. The results showed that G ± chemotherapy significantly improved the CRR in patients with a high tumor burden (*P*=0.002). Notably, the GALLIUM study did not specifically investigate the subgroup effects of different treatment regimens in high tumor burden MZL patients (12). Our study preliminarily reveals the potential advantage of G in treating high tumor burden MZL, suggesting superior efficacy. This finding has important implications for clinical practice. For patients with high tumor burden MZL, G combined with chemotherapy may offer a higher CRR and should be considered by clinicians when devising treatment plans. In contrast, for patients with low tumor burden, the efficacy of the two monoclonal antibodies appears similar, allowing for a balanced choice based on the patient’s specific condition and the side effect profiles of the drugs. However, it is undeniable that the small sample size of our study limits the generalizability of the results. In the future, we aim to expand the sample size and extend the follow-up period to more accurately assess the efficacy of G in this specific patient population.

### 4.3 Prognostic analysis

Previous studies have established POD24 as a critical prognostic factor for MZL patients (28). Conconi et al. (29) analyzed patients from the IELSG-19 study who received immunochemotherapy and found that 18% (69/386) experienced a POD24 event. These patients had a 10-year OS of 64%, compared to 85% in the control group who did not experience POD24. Similarly, in the NF10 observational study (30), 18% (59/321) of MZL patients experienced POD24, leading to a 3-year OS of 53%, whereas patients without POD24 had a 3-year OS of 95%. In our study, 11.6% (17/147) of patients experienced POD24, a lower incidence than reported in the aforementioned studies. However, the median survival for patients with POD24 in our cohort was only 19.7 months, with a 3-year OS of just 37.6%. In contrast, patients who did not experience POD24 had a 3-year OS of 97.3%. This finding underscores the severe impact of POD24 on the survival of MZL patients. This study utilized nomogram analysis to identify key factors influencing OS, with a particular emphasis on the significant impact of POD24. However, due to the insufficient sample size, the calibration curve exhibited some bias. This finding suggests that future research should focus on increasing the sample size and further validating the predictive performance of the model to enhance its clinical applicability.

In recent studies, the MZL-IPI has been established as the first prognostic index for all subtypes of newly diagnosed symptomatic MZL (31). An external validation cohort from the United States included 353 MZL patients, with 94 (27%) classified as low-risk, 192 (54%) as intermediate-risk, and 67 (19%) as high-risk. After a median follow-up of 77.8 months, the 5-year PFS rates for these groups were 69%, 57%, and 45%, respectively (*P*=0.0018), while the 5-year OS rates were 93%, 84%, and 69%, respectively (*P*<0.001). In contrast to the aforementioned study, our research found that only the low-risk group had significantly better PFS (*P*=0.025) and OS (*P*=0.040) compared to the high-risk group according to the MZL-IPI. Potential reasons for this discrepancy include our relatively smaller sample size. We plan to address this by increasing the sample size and extending the follow-up period in future studies to more accurately validate the clinical utility of the MZL-IPI. Additionally, unlike the U.S. study, all patients in our research received anti-CD20 monoclonal antibody therapy, whereas some patients in the U.S. study did not. Consequently, the 5-year PFS and OS rates in our study were numerically higher. Nonetheless, the MZL-IPI, as the first prognostic index encompassing all MZL subtypes, provides critical guidance for clinicians in developing treatment strategies. Effective risk stratification based on the MZL-IPI allows clinicians to tailor individualized treatment plans, potentially improving long-term survival outcomes. Future research should further explore the application of MZL-IPI in different therapeutic contexts, including novel targeted therapies and immunotherapies. Additionally, studies could investigate integrated prognostic models that combine MZL-IPI with other biomarkers to enhance the predictive accuracy for MZL patient outcomes.

### 4.4 Safety analysis

The results of the GALLIUM study indicate that the proportion of patients experiencing Grade 3-5 AEs was higher in the group receiving G-chemotherapy compared to those receiving R-chemotherapy (86.1% vs. 77.4%), suggesting that G-chemotherapy has lower tolerability than R-chemotherapy (12). In our study, the incidence of Grade 3-4 AEs was also higher in the G ± chemotherapy group compared to the R ± chemotherapy group (52.8% vs. 31.5%). Additionally, two patients in the G ± chemotherapy group died from COVID-19 infection within 1-6 months after completing treatment. According to the AEs assessment criteria in the GALLIUM study, these were classified as Grade 5 serious AEs. This phenomenon may be related to the stronger ADCC effect associated with G-chemotherapy, which can lead to more significant immunosuppressive effects (32). Therefore, we need to pay closer attention to the severe immunosuppressive consequences that may arise from G ± chemotherapy regimens. In summary, although G-chemotherapy demonstrates better efficacy in certain aspects, the risk of immunosuppression it induces requires significant clinical attention. By optimizing treatment strategies and enhancing monitoring, we can improve efficacy while maximizing patient safety.

## 5 Limitations

This study has several limitations: (1) As a retrospective study, it inherently suffers from limitations such as selection bias, ambiguous temporal relationships, and restricted generalizability; (2) The wide time span of sample collection poses challenges in data retrieval for certain cases; (3) Regarding the use of novel anti-CD20 monoclonal antibody, the limited application duration and small sample size necessitate future studies to expand the sample size and extend the follow-up period.

## 6 Conclusion

MZL, commonly seen in middle-aged and elderly individuals, is a specific indolent B-cell lymphoma, with MALT lymphoma being the most prevalent pathological type. Achieving CR after induction therapy significantly prolongs the survival of MZL patients. Compared to R ± chemotherapy, G ± chemotherapy has achieved higher CRR in high tumor burden MZL, particularly in patients with a high tumor burden. In the era of immunotherapy, POD24 remains an independent prognostic factor for MZL.

## Data Availability

The raw data supporting the conclusions of this article will be made available by the authors, without undue reservation.
