# Supplementary material for: Characteristics, efficacy, and prognosis analysis of newly diagnosed marginal zone lymphoma
Source: Front Immunol. 2024 Sep 23;15:1466859. doi: 10.3389/fimmu.2024.1466859 (PMC11456499; doi:10.3389/fimmu.2024.1466859)
Supplement: Supplementary file 1 [file Table1.docx]

Supplementary Table 1: Univariate and Multivariate Analysis of PFS

| Characteristics |  | Univariate Analysis | | Multivariate analysis | |
| --- | --- | --- | --- | --- | --- |
|  |  | HR (95%CI) | *P* | HR (95%CI) | *P* |
| Sex | Female | reference |  |  |  |
|  | Male | 2.057 (0.924-4.581) | 0.078 |  |  |
| Age | ＜60 years | reference |  |  |  |
|  | ≥60 years | 1.303 (0.611-2.778) | 0.493 |  |  |
| MZL subtype | MALT/NMZL | reference |  |  |  |
|  | NMZL/DissMZL | 1.867 (0.854-4.080) | 0.118 |  |  |
| Ann Arbor stage | Ⅰ-Ⅱ | reference |  |  |  |
|  | Ⅲ-Ⅳ | 2.032 (0.820-5.036) | 0.126 |  |  |
| MZL-IPI | 0 | reference |  |  |  |
|  | 1-2 | 1.541 (0.628-3.783) | 0.345 |  |  |
|  | 3-5 | 3.382 (1.071-10.676) | 0.038 | 0.457 (0.066-3.162) | 0.428 |
| ECOG | ＜2 | reference |  |  |  |
|  | ≥2 | 1.909 (0.825-4.417) | 0.131 |  |  |
| B symptoms | No | reference |  |  |  |
|  | Yes | 2.917 (1.351-6.298) | 0.006 | 1.673 (0.524-5.340) | 0.385 |

Supplementary Table 1 (Continued)

| Characteristics |  | Univariate Analysis | | Multivariate analysis | |
| --- | --- | --- | --- | --- | --- |
|  |  | HR (95%CI) | *P* | HR (95%CI) | *P* |
| LDH | ≤UNL | reference |  |  |  |
|  | ＞UNL | 1.589 (0.600-4.204) | 0.351 |  |  |
| ALC | ≥1$\times$10^9^/L | reference |  |  |  |
|  | ＜1$\times$10^9^/L | 0.562 (0.169-1.867) | 0.347 |  |  |
| HB | ≥12 g/dL | reference |  |  |  |
|  | ＜12 g/dL | 2.293 (1.071-4.908) | 0.033 | 2.326 (0.738-7.334) | 0.150 |
| PLT | ≥100$\times$10^9^/L | reference |  |  |  |
|  | ＜100$\times$10^9^/L | 2.761 (1.160-6.569) | 0.022 | 1.625 (0.534-4.942) | 0.393 |
| Bone marrow involvement | No | reference |  |  |  |
|  | Yes | 2.049 (0.960-4.373) | 0.064 |  |  |
| High tumor burden | No | reference |  |  |  |
|  | Yes | 3.180 (1.444-7.004) | 0.004 | 1.853 (0.529-6.486) | 0.335 |
| INV-assessed response | CR | reference |  |  |  |
|  | non-CR | 3.020 (1.356-6.729) | 0.007 | 3.250 (1.409-7.500) | 0.006 |

PFS: progression-free survival; MZL: marginal zone lymphoma; MALT: mucosa-associated lymphoid tissue; NMZL: nodal MZL; SMZL: splenic MZL; DissMZL: disseminated MZL; MZL-IPI: MZL-international prognostic index; ECOG: Eastern Cooperative Oncology Group; LDH: lactate dehydrogenase; ALC: absolute lymphocyte count; HB: hemoglobin; PLT: platelet; UNL: Upper Normal Limit; INV: investigator; CR: complete response.

Supplementary Table 2: Univariate and Multivariate Analysis of OS

| Characteristics |  | Univariate Analysis | | Multivariate analysis | |
| --- | --- | --- | --- | --- | --- |
|  |  | HR (95%CI) | *P* | HR (95%CI) | *P* |
| Sex | Female | reference |  |  |  |
|  | Male | 4.034 (1.138-14.305) | 0.031 | 2.914 (0.781-10.875) | 0.111 |
| Age | ＜60 years | reference |  |  |  |
|  | ≥60 years | 1.361 (0.493-3.760) | 0.552 |  |  |
| MZL subtype | MALT/NMZL | reference |  |  |  |
|  | NMZL/Diss MZL | 2.034 (0.723-5.720) | 0.178 |  |  |
| Ann Arbor stage | Ⅰ-Ⅱ | reference |  |  |  |
|  | Ⅲ-Ⅳ | 3.846 (0.868-17.049) | 0.076 |  |  |
| MZL-IPI | 0 | reference |  |  |  |
|  | 1-2 | 2.175 (0.588-8.042) | 0.244 |  |  |
|  | 3-5 | 4.827 (0.972-23.967) | 0.054 |  |  |
| ECOG | ＜2 | reference |  |  |  |
|  | ≥2 | 2.213 (0.747-6.557) | 0.152 |  |  |
| B symptoms | No | reference |  |  |  |
|  | Yes | 2.683 (0.954-7.548) | 0.061 |  |  |

Supplementary Table 2 (Continued)

| Characteristics |  | Univariate Analysis | | Multivariate analysis | |
| --- | --- | --- | --- | --- | --- |
|  |  | HR (95%CI) | *P* | HR (95%CI) | *P* |
| LDH | ≤UNL | reference |  |  |  |
|  | ＞UNL | 1.797 (0.506-6.388) | 0.365 |  |  |
| ALC | ≥1$\times$10^9^/L | reference |  |  |  |
|  | ＜1$\times$10^9^/L | 0.752 (0.170-3.335) | 0.708 |  |  |
| HB | ≥12 g/dL | reference |  |  |  |
|  | ＜12 g/dL | 2.615 (0.929-7.359) | 0.069 |  |  |
| PLT | ≥100$\times$10^9^/L | reference |  |  |  |
|  | ＜100$\times$10^9^/L | 3.911 (1.329-11.508) | 0.013 | 0.753 (0.208-2.719) | 0.665 |
| Bone marrow involvement | No | reference |  |  |  |
|  | Yes | 3.384 (1.198-9.558) | 0.021 | 3.321 (0.967-11.406) | 0.057 |
| High tumor burden | No | reference |  |  |  |
|  | Yes | 2.667 (0.943-7.546) | 0.064 |  |  |
| POD24 | No | reference |  |  |  |
|  | Yes | 24.596 (8.233-73.479) | ＜0.001 | 22.544 (6.390-79.541) | ＜0.001 |

Supplementary Table 2 (Continued)

| Characteristics |  | Univariate Analysis | | Multivariate analysis | |
| --- | --- | --- | --- | --- | --- |
|  |  | HR (95%CI) | *P* | HR (95%CI) | *P* |
| INV-assessed response | CR | reference |  |  |  |
|  | non-CR | 9.412 (2.122-41.740) | 0.003 | 5.1766 (1.075-24.934) | 0.040 |

OS: overall survival; MZL: marginal zone lymphoma; MALT: mucosa-associated lymphoid tissue; NMZL: nodal MZL; SMZL: splenic MZL; DissMZL: disseminated MZL; MZL-IPI: MZL-international prognostic index; ECOG: Eastern Cooperative Oncology Group; LDH: lactate dehydrogenase; ALC: absolute lymphocyte count; HB: hemoglobin; PLT: platelet; POD24: disease progression or death within 24 months of initial treatment; UNL: Upper Normal Limit; INV: investigator; CR: complete response.


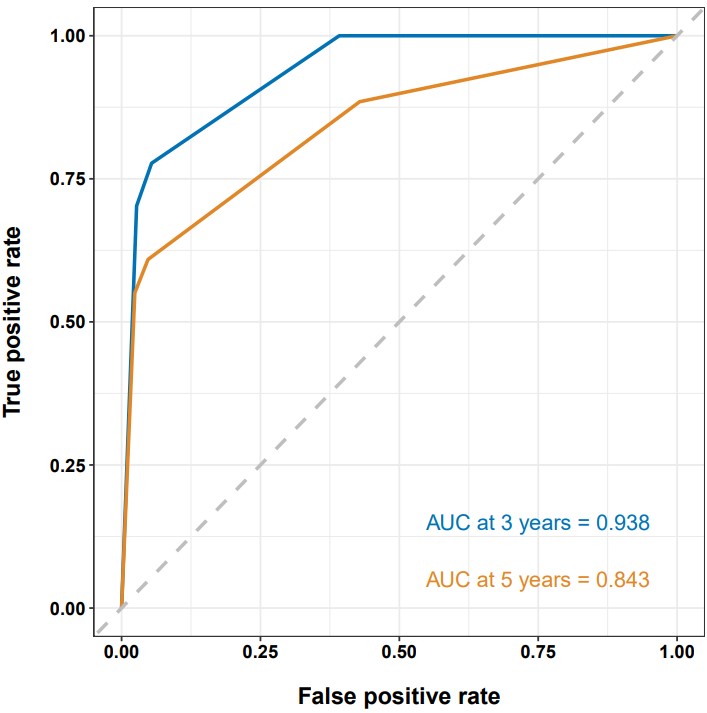


Supplementary Figure 1: Nomogram ROC Curve


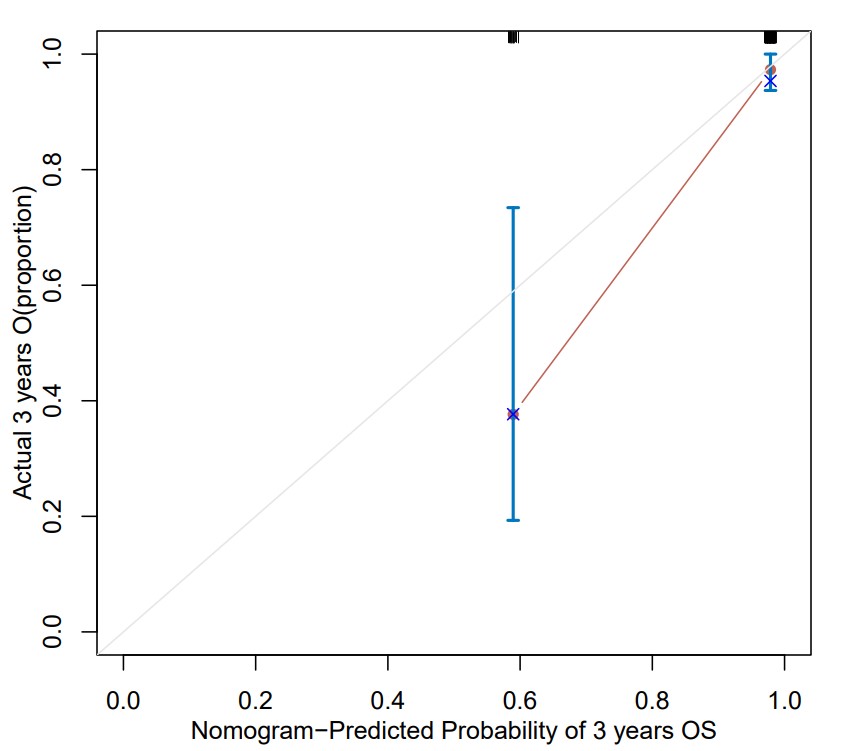


Supplementary Figure 2: Nomogram Calibration Curve (3 years OS)


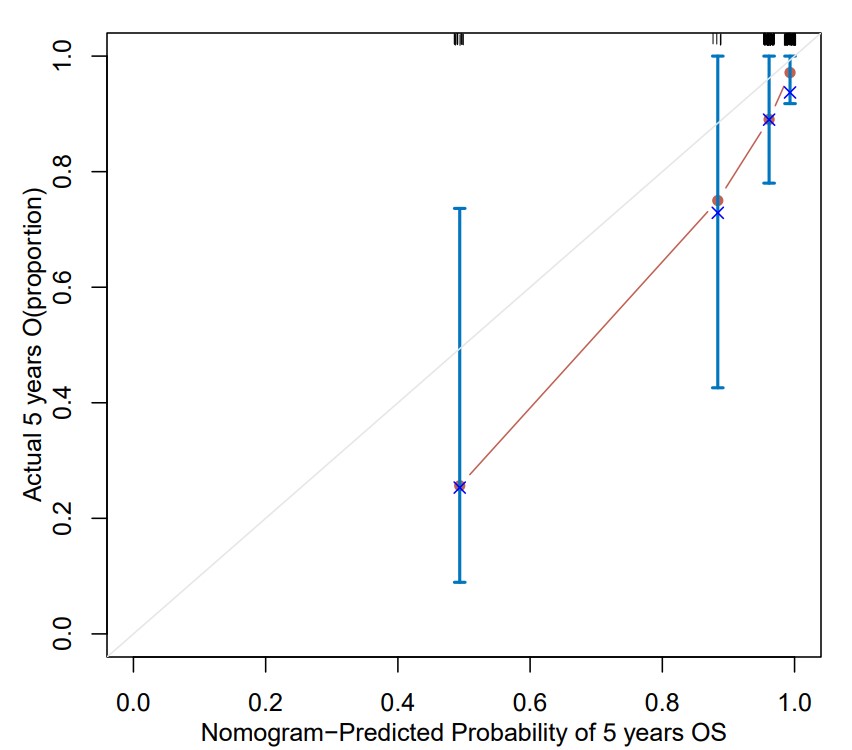


Supplementary Figure 3: Nomogram Calibration Curve (5 years OS)
